# Safety and effectiveness of tepotinib in patients with unresectable, advanced or recurrent non-small cell lung cancer with *METex14* skipping alterations: post-marketing surveillance in Japan

**DOI:** 10.1093/jjco/hyaf099

**Published:** 2025-06-20

**Authors:** Terufumi Kato, Tatsuya Ogura, Masashi Sato, Risa Kojima, Bingbing Song, Eisuke Horii, Kazuhiko Nakagawa

**Affiliations:** Department of Thoracic Oncology, Kanagawa Cancer Center, 2-3-2 Nakao, Asahi-ku, Yokohama, Kanagawa 241-8515, Japan; Clinical Research Center, Tokyo Metropolitan Cancer and Infectious Diseases Center Komagome Hospital, 3-18-22 Honkomagome, Bunkyo-ku, Tokyo 113-8677, Japan; PMS Planning & Strategy, Merck Biopharma Co, Ltd. (an affiliate of Merck KGaA), 1.3.1 Azabudai, Minato-ku, Tokyo 106-0041, Japan; Global Research & Development, Clinical Measurement Sciences, Biostatistics, Merck Biopharma Co, Ltd. (an affiliate of Merck KGaA), 1-3-1 Azabudai, Minato-ku, Tokyo 106-0041, Japan; Global Patient Safety Japan, Merck Biopharma Co, Ltd. (an affiliate of Merck KGaA), 1-3-1 Azabudai, Minato-ku, Tokyo 106-0041, Japan; Global Development Operations Japan, R&D, Merck Biopharma Co, Ltd. (an affiliate of Merck KGaA), 1-3-1 Azabudai, Minato-ku, Tokyo 106-0041, Japan; Oncology Medical Affairs, Merck Biopharma Co, Ltd. (an affiliate of Merck KGaA), 1-3-1 Azabudai, Minato-ku, Tokyo 106-0041, Japan; Department of Medical Oncology, Kindai University Faculty of Medicine, 377-2 Ohno-higashi, Osaka-Sayama, Osaka 589-8511, Japan

**Keywords:** Japanese, MET receptor tyrosine kinase, non-small cell lung cancer, tepotinib, post-marketing surveillance

## Abstract

**Objective:**

Tepotinib has shown clinical benefits in patients with non-small cell lung cancer (NSCLC) harboring MET exon 14 (*METex14*) skipping alterations. The objective of this post-marketing surveillance (PMS) was to assess the safety and effectiveness of tepotinib in general clinical practice in Japan.

**Methods:**

This multicenter, non-interventional PMS included patients with unresectable, advanced, or recurrent NSCLC with *METex14* skipping alterations who received at least one dose of tepotinib. The primary endpoint was the incidence of adverse drug reactions (ADRs) identified as safety specifications (SS) for tepotinib according to the Japanese Risk Management Plan (ADRs of SS); i.e. interstitial lung disease (ILD), fluid retention, hepatic function disorder, renal impairment, and QT interval prolongation (classified by preferred term). The secondary endpoints were ADRs of SS in patient subgroups and the effectiveness of tepotinib in clinical practice. In total, 147 patients were included (median age 72.0 years).

**Results:**

The incidence of ILD, fluid retention, hepatic function disorder, and renal impairment was 7.5%, 47.6%, 13.6%, and 36.1%, respectively; incidence of grade ≥3 ADRs of SS were 3.4%, 9.5%, 4.8%, and 2.7%, respectively. No instances of QT interval prolongation occurred. Four patients (2.7%) died due to ILD. The objective response rate was 51.0% [95% confidence interval (CI) 42.7, 59.3] and disease control rate was 77.6% (95% CI 69.9, 84.0).

**Conclusions:**

Tepotinib was generally well tolerated and effective in patients with NSCLC and *METex14* skipping alterations in general clinical practice in Japan; no new safety issues or concerns were identified.

## Teaser

The results of this post-marketing surveillance show that tepotinib is generally well tolerated and effective in patients with NSCLC with *METex14* skipping alterations in general clinical practice in Japan.

## Introduction

Lung cancer is the leading cause of cancer-related deaths worldwide [1]. Non-small cell lung cancer (NSCLC) accounts for ~80% of lung cancer cases [2, 3].

MET is a receptor tyrosine kinase involved in a variety of cellular processes [4]. MET exon 14 (*METex14*) skipping alterations result in oncogenic MET signaling, promoting tumor cell proliferation, survival, migration, and invasion [4, 5]. *METex14* skipping alterations tend to be the primary oncogenic driver and are present in 0.6%–6.6% of patients with NSCLC [5–9]. Patients with NSCLC who harbor *METex14* skipping alterations are usually elderly and have a poor prognosis [10]. Therefore, there was an unmet medical need for effective treatment options in this patient population.

Tepotinib is a selective and potent MET inhibitor that can be administered orally [11]. In the open-label, single-arm phase II VISION study (NCT02864992), tepotinib 500 mg once daily (450 mg active moiety) was associated with a rapid and durable response to treatment, regardless of age or previous treatment [12–14]. The safety profile of tepotinib in VISION was manageable and peripheral edema was the most common treatment-related adverse event (AE) [12, 13, 15]. In 2018, tepotinib received the Sakigake (fast-track) designation from the Ministry of Health, Labor, and Welfare (MHLW) of Japan [11, 16]. In March 2020, the MHLW of Japan granted tepotinib full approval for the treatment of patients with unresectable, advanced, or recurrent NSCLC with *METex14* skipping alterations [17], making tepotinib the first MET inhibitor to be approved for this indication in any country [17]. A subset analysis of 38 Japanese patients from the phase II VISION study demonstrated robust and durable clinical efficacy in Japanese patients; an increase in blood creatinine and peripheral edema were the most common AEs, and were considered manageable [18, 19]. Based on data from the VISION study, the Japan Lung Cancer Society strongly recommended tepotinib for the treatment of NSCLC with *METex14* skipping alterations [20].

Since the VISION study enrolled a limited number of Japanese patients, implementation of all patient surveillance (post-marketing surveillance; PMS) was required as a condition of approval. The objective of this PMS was to further characterize the safety and effectiveness of tepotinib in the treatment of patients with NSCLC with *METex14* skipping alterations in the general clinical practice setting in Japan. The PMS was designed based on data from the VISION study at the time of approval application in Japan, and was conducted to confirm that there were no data that greatly diverged from the data at the time of approval in Japanese patients.

## Patients and methods

### Study design and patient population

This was a multicenter, non-interventional, drug use-results survey of patients with unresectable, advanced or recurrent NSCLC with *METex14* skipping alterations receiving tepotinib 500 mg once daily in general clinical practice in Japan. This PMS was conducted as an additional pharmacovigilance activity specified in the Japanese Risk Management Plan (J-RMP) for tepotinib. All patients who received at least one dose of tepotinib between 1 June 2020 and 30 November 2020 were eligible. In addition, patients who started to receive tepotinib on or after 1 December 2020, whose enrolment was completed by 31 March 2021, were eligible for this PMS. Investigators at each participating clinical site recorded patient information using electronic or paper case report forms (CRFs). We planned to collect patient data over a 52-week period from the first dose of tepotinib (observational period).

According to the Ministerial Ordinance on Good Post-marketing Study Practice (GPSP), approval from independent ethics committees and institutional review boards was not required. However, the protocol and other relevant documents were provided to all participating sites, and approval was obtained if required according to the rules at each site. The protocol and other relevant documents were submitted to the Pharmaceuticals and Medical Devices Agency of Japan. Although data from all eligible patients were recorded, only those patients who consented to the publication of their data were included in the analyses.

### Endpoints

The primary endpoint was the incidence of interstitial lung disease (ILD), fluid retention, hepatic function disorder, renal impairment, and QT interval prolongation. These five adverse drug reactions (ADRs) were identified having important safety risks associated with tepotinib, according to the safety specifications in the J-RMP; only these ADRs of safety specifications (classified by preferred term) were monitored in this PMS and no data on the incidence of other ADRs outside of these ADRs were collected. An ADR was defined as an untoward medical occurrence for which a causal relationship with tepotinib was at least a reasonable possibility. The incidence of the ADRs of safety specifications were analyzed by patient subgroups [age and Eastern Cooperative Oncology Group performance status (ECOG PS)]. ADRs were classified according to the Japanese version of the Medical Dictionary for Regulatory Activities (MedDRA/J), version 25.1, using the system-organ-class and preferred term nomenclature.

The secondary endpoints in this analysis were the best overall response rates, the objective response rate (ORR), duration of response (DOR), and progression-free survival (PFS) and its corresponding rates at 6 and 12 months. ORR was defined as the proportion of patients whose best overall response was complete response (CR) or partial response (PR). DOR was defined as the time from first achieving CR or PR until progression of disease (PD) or death due to any cause, or until the end of the observation period. Response was assessed by the treating oncologist with reference to the Japanese version of the Response Evaluation Criteria in Solid Tumors (RECIST), version 1.1. Disease control rate (DCR) was also assessed and was defined as the proportion of patients whose best overall response was CR, PR, or stable disease (SD).

### Statistical analyses

From a feasibility standpoint, and because *METex14* skipping alteration is a rare condition, the target number of patients was set as 100. Both the safety and effectiveness analysis populations included patients in the “finalized CRF population”, whose CRFs had been collected and the data locked (database lock date—13 November 2023) and whose consent was obtained for publication.

Descriptive statistics were used to assess baseline characteristics, with categorical variables presented as overall number and proportion of patients and continuous variables presented as median and range. In the safety analysis, the overall number and proportion of patients with ADRs of safety specifications, as well as the number and proportion of patients with ADRs of safety specifications categorized by severity, seriousness, and outcome, were calculated. The cumulative incidence of ADRs of safety specifications were summarized using the number and proportion of patients with corresponding two-sided exact Clopper–Pearson 95% confidence intervals (CIs). The time to onset and time to resolution of ADRs of safety specifications were calculated and presented as medians and their corresponding ranges. Resolution was defined as the sum of recovered and resolving ADRs. In the effectiveness analysis, the number and proportion of patients with each type of best overall response were calculated. In addition, the proportion of patients with an ORR or DCR, as well as the corresponding two-sided exact Clopper–Pearson 95% CIs were calculated. Kaplan–Meier estimates of PFS and overall survival (OS) rates at 6 and 12 months after the first dose of tepotinib, as well as median PFS and OS, were calculated, along with two-sided 95% CIs.

Statistical analyses were conducted using SAS software, version 9.4 (Cary, NC, USA).

## Results

### Patients and treatment

**Figure 1:**
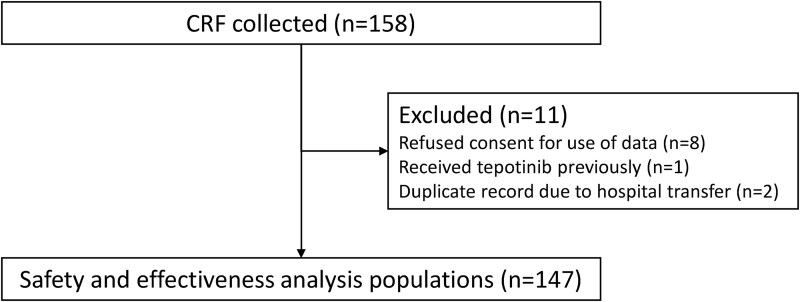
Patient disposition. CRF, case report form.

CRFs were collected from 158 patients (Fig. 1). Of these, 11 patients were excluded from the analyses (refused consent for the use of data, *n* = 8; received tepotinib previously, *n* = 1; duplicate record due to hospital transfer, *n* = 2). The remaining 147 patients were included in both the safety and the effectiveness analysis populations.

Baseline patient characteristics are shown in Table 1. The median age of included patients was 72.0 years; 27 patients (18.4%) were aged <65 years, 55 (37.4%) were aged 65 to <75 years, and 65 (44.2%) were aged ≥75 years. Most patients had an ECOG PS of 0–1 (*n* = 112, 76.2%), while 31 patients (21.1%) had an ECOG PS of 2–4. Adenocarcinoma was the most common histological type (*n* = 112, 76.2%), followed by sarcomatoid carcinoma (*n* = 13, 8.8%) and squamous cell carcinoma (*n* = 8, 5.4%). Tepotinib was administered as first-line therapy in 71 patients (48.3%), as second-line therapy in 30 (20.4%), as third-line therapy in 13 (8.8%), and as fourth- or later-line therapy in 33 (22.4%).

**Table 1:** Baseline patient characteristics.

**Characteristic**	***n* = 147**
Age, years, median (range)	72.0 (42–90)
Age, *n* (%)	
<65 years	27 (18.4)
65 to <75 years	55 (37.4)
75 to <85 years	50 (34.0)
≥85 years	15 (10.2)
Sex, *n* (%)	
Male	79 (53.7)
Female	68 (46.3)
Smoking history, *n* (%)	
Never	69 (46.9)
Current	7 (4.8)
Former	71 (48.3)
ECOG PS, *n* (%)	
0	36 (24.5)
1	76 (51.7)
2	17 (11.6)
3	13 (8.8)
4	1 (0.7)
Unknown	4 (2.7)
Histology, *n* (%)	
Adenocarcinoma	112 (76.2)
Sarcomatoid carcinoma	13 (8.8)
Squamous cell carcinoma	8 (5.4)
Other^a^	14 (9.5)
Treatment line, *n* (%)	
First	71 (48.3)
Second	30 (20.4)
Third	13 (8.8)
Fourth or later	33 (22.4)
Prior treatment history of NSCLC^b^, *n* (%)	
Chemotherapy	75 (51.0)
ICI	57 (38.8)
Crizotinib	5 (3.4)
Other^c^	23 (15.6)
Concurrent complications^b^, *n* (%)	
Renal impairment	28 (19.0)
Hepatic function disorder	9 (6.1)
Allergy	8 (5.4)
ILD	7 (4.8)
PD-L1 status^d^, *n* (%)	
Not tested	14 (9.5)
Tested	133 (90.5)
Positive	106 (79.7)
Negative	27 (20.3)

ECOG PS, Eastern Cooperative Oncology Group performance status; ICI, immune checkpoint inhibitor; ILD, interstitial lung disease; NSCLC, non-small cell lung cancer; PD-L1, programmed death-ligand 1.

^a^Includes nonsmall cell carcinoma, not otherwise specified (*n* = 11, 7.5%) and adenosquamous carcinoma (*n* = 3; 2.0%).

^b^Patients could have multiple conditions.

^c^Includes other oncology drugs, gefitinib, osimertinib mesylate, bevacizumab, and ramucirumab.

^d^Calculated on a reported basis, regardless of the positivity rate.

“–” indicate that the value is zero.

The characteristics of patients were similar among the age (Supplementary Table S1) and ECOG PS (Supplementary Table S2) subgroups.

The median treatment duration was 4.8 months (range 0.1–23.7). A total of 114 patients (77.6%) discontinued tepotinib treatment during the observational period. The most common reasons for discontinuation were disease progression (*n* = 72, 49.0%), followed by ADRs of safety specifications (*n* = 25, 17.0%).

### Safety

ADRs of safety specifications described in the J-RMP are reported in this section. ADRs of any grade occurred in 106 patients (72.1%) and grade ≥3 ADRs occurred in 26 patients (17.7%; Table 2).

**Table 2:** Incidence of adverse drug reactions of safety specifications.

**ADR, *n* (%)**	***n* = 147**	
	**Any grade**	**Grade ≥3**
Any	106 (72.1)	26 (17.7)
ILD	11 (7.5)	5 (3.4)
ILD	8 (5.4)	4 (2.7)
Pneumonitis	2 (1.4)	–
Radiation pneumonitis	1 (0.7)	1 (0.7)
Fluid retention	70 (47.6)	14 (9.5)
Peripheral edema	57 (38.8)	7 (4.8)
Pleural effusion	7 (4.8)	2 (1.4)
Systemic edema	6 (4.1)	4 (2.7)
Hypoalbuminemia	1 (0.7)	1 (0.7)
Localized edema	1 (0.7)	–
Periorbital edema	1 (0.7)	–
Hepatic function disorder	20 (13.6)	7 (4.8)
Hepatic function abnormal	9 (6.1)	3 (2.0)
ALT increased	7 (4.8)	2 (1.4)
AST increased	6 (4.1)	1 (0.7)
Ascites	1 (0.7)	1 (0.7)
Hepatobiliary disease	1 (0.7)	1 (0.7)
Drug-induced liver injury	1 (0.7)	–
Renal impairment	53 (36.1)	4 (2.7)
Blood creatinine increased	32 (21.8)	2 (1.4)
Impaired renal function	14 (9.5)	2 (1.4)
Renal disorder	5 (3.4)	–
Acute kidney injury	3 (2.0)	–
QT interval prolongation	–	–

ADRs of safety specifications were classified according to MedDRA/J, version 25.1.

ADRs, adverse drug reactions; ALT, alanine aminotransferase; AST, aspartate aminotransferase; ILD, interstitial lung disease; MedDRA/J, Japanese version of Medical Dictionary for Regulatory Activities.

“–” indicates that the value is zero.

**Figure 2:**
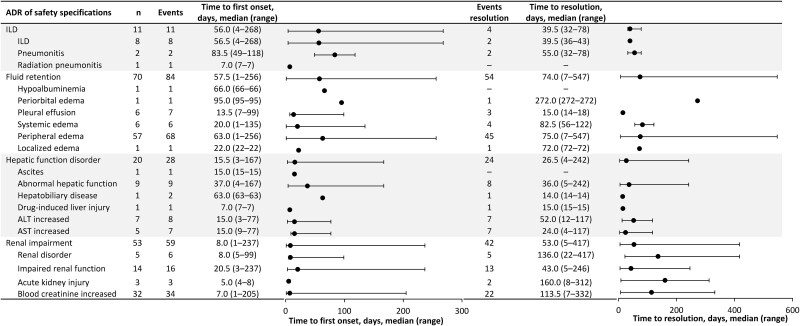
Time to onset and resolution^a^ of adverse drug reactions of safety specifications. ^a^Resolution was defined as the sum of recovered and resolving. ADR, adverse drug reaction; ALT, alanine aminotransferase; AST, aspartate aminotransferase; ILD, interstitial lung disease; n, number of patients with the event. “–” indicates that the value is zero.

ILD ADRs of any grade occurred in 11 patients (7.5%) in this PMS and, of these, seven patients had ILD as a pre-existing condition or concurrent complication. ILD ADRs of grade ≥3 occurred in five patients (3.4%; Table 2). Serious ILD ADRs occurred in seven patients (4.8%) and nonserious ILD ADRs in four patients (2.7%; Supplementary Table S3). ILD ADRs occurred in six of the 57 patients (10.5%) who had previously received immune checkpoint inhibitor therapy and in five of the 90 patients (5.6%) who had not previously received immune checkpoint inhibitor therapy. By the end of the observation period, four patients (2.7%) who had ILD ADRs were resolving, one (0.7%) had recovered with sequelae, two patients (1.4%) had not recovered from ILD, and four patients (2.7%) who had ILD ADRs had died. When ADRs were classified by preferred term, three of the patients who died had ILD and one patient had radiation pneumonitis. All four patients who died had active ILD at the start of tepotinib treatment. The median time to onset of ILD ADRs from the start of tepotinib treatment was 56.0 days and the median time from onset to resolution was 39.5 days (Fig. 2). ILD ADRs did not influence tepotinib treatment in two patients, while in the remaining nine patients they led to treatment interruption (two patients), dose reduction (one patient), or discontinuation (six patients).

Fluid retention ADRs of any grade occurred in 70 patients (47.6%) and of grade ≥3 in 14 patients (9.5%; Table 2). Serious fluid retention ADRs occurred in nine patients (6.1%) and nonserious fluid retention ADRs in 61 patients (41.5%; Supplementary Table S3). By the end of the observation period, 16 patients (10.9%) who had fluid retention ADRs had recovered, 26 (17.7%) were resolving, and fluid retention ADRs remained unrecovered in 29 (19.7%; duplicate cases existed). The median time to onset of fluid retention ADRs from the start of tepotinib treatment was 57.5 days and the median time from onset to resolution was 74.0 days (Fig. 2). Fluid retention ADRs did not influence tepotinib treatment in 26 patients, while in the remaining 44 patients they led to treatment interruption (25 patients), dose reduction (29 patients), or discontinuation (14 patients; duplicate cases existed).

When classified by preferred term, peripheral edema was the most common fluid retention ADR (38.8%). Among the 57 patients who experienced peripheral edema, the specific sites of 68 occurrences of edema (number of occurrences in brackets) were: the extremities (27), lower extremities (20), lower legs (14), and upper extremities (1); no information on the specific sites of edema was obtained for six occurrences. Curative actions taken after the occurrences of peripheral edema related to tepotinib included discontinuation (eight occurrences), dose reduction (26), treatment interruption (30), or no action (30; duplicate cases existed). One occurrence of peripheral edema occurred after drug discontinuation. Other curative actions against peripheral edema not related to tepotinib were taken in 36 occurrences and not taken in 32. There were 36 occurrences of curative actions involving drug administration, which included furosemide (28 occurrences), spironolactone (6), kampo drugs (3), prednisolone (2), tolvaptan (2), and torsemide, azosemide, and trichlormethiazide (one occurrence each). Other curative actions taken against peripheral edema were elastic stockings (four occurrences), lymphatic massage (1), and dietary salt restriction in one occurrence (duplicate cases existed). Dose modification and/or diuretic-based treatment tended to resolve peripheral edema (resolution rates of 50.0%–94.7%) compared with no intervention (38.5%); see Table 3 for details of resolution rate of peripheral edema based on dose change and diuretic-based therapy.

**Table 3:** Resolution rate of peripheral edema based on dose change and diuretic-based therapy.

**Tepotinib dose change**	**Diuretic-based therapy**	**Resolution** ^a^ **/total number of events (%)**	**95% CI**	**Grade breakdown of peripheral edema events, *n* (%)**		
				**Grade 1**	**Grade 2**	**Grade 3**
Yes	Yes	8/16 (50.0)	24.7–75.3	3 (18.8)	8 (50.0)	5 (31.3)
Yes	None^b^	18/19 (94.7)	74.0–99.9	7 (36.8)	11 (57.9)	1 (5.3)
None	Yes	8/14 (57.1)	28.9–82.3	2 (14.3)	12 (71.4)	0 (0)
None	None^c^	5/13 (38.5)	13.9–68.4	7 (53.9)	5 (38.5)	1 (7.7)

CI, confidence interval.

^a^Resolution was defined as the sum of recovered and resolving.

^b^Other than diuretics, corticosteroids (resolution rate: 1/1) and elastic stockings (2/2) were administered, but these were excluded from this analysis.

^c^Other than diuretics, treatment was performed with traditional Chinese medicine (1/1), leg elevation and elastic stockings (0/1), and dietary salt restriction (1/1), but excluded from this analysis.

**Figure 3:**
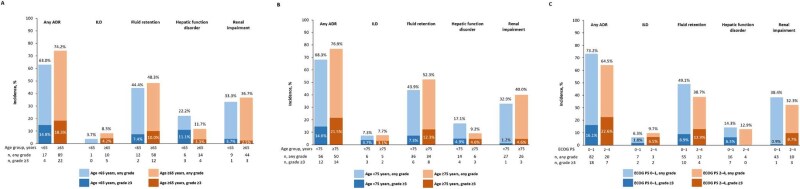
Incidence of adverse drug reactions of safety specifications by age (a) <65 and ≥65 years and (b) <75 and ≥75 years; and by (c) Eastern Cooperative Oncology Group performance status. ADR, adverse drug reaction; ECOG PS, Eastern Cooperative Oncology Group performance status; ILD, interstitial lung disease; n, number of patients with the event.

Hepatic function disorder ADRs of any grade occurred in 20 patients (13.6%) and of grade ≥3 in seven patients (4.8%; Table 2). Most hepatic function disorders were associated with elevated hepatic enzymes (preferred terms: hepatic function abnormal, alanine transaminase/aspartate transaminase increased). Serious hepatic function disorder ADRs occurred in two patients (1.4%) and nonserious hepatic function disorder ADRs in 18 patients (12.2%; Supplementary Table S3). By the end of the observation period, 10 patients (6.8%) who had hepatic function disorder ADRs had recovered, six (4.1%) were resolving, and in four patients (2.7%), hepatic function disorder ADRs remained unrecovered. The median time from the start of tepotinib treatment to onset of hepatic function disorder ADRs was 15.5 days and the median time from onset to resolution was 26.5 days (Fig. 2). Hepatic function disorder ADRs did not influence tepotinib treatment in seven patients, while in the remaining 13 patients they led to treatment interruption (nine patients), dose reduction (nine patients), or discontinuation (two patients; duplicate cases existed).

Renal impairment ADRs of any grade occurred in 53 patients (36.1%) and of grade ≥3 in four patients (2.7%; Table 2). Serious renal impairment ADRs occurred in three patients (2.0%) and nonserious renal impairment ADRs in 50 patients (34.0%; Supplementary Table S3). By the end of the observation period, 20 patients (13.6%) who had renal impairment ADRs had recovered, 17 (11.6%) were resolving, one (0.7%) had recovered with sequelae, and in 15 patients (10.2%), renal impairment ADRs remained unrecovered. The median time from the start of tepotinib treatment to onset of renal impairment ADRs was 8.0 days and the median time from onset to resolution was 53.0 days (Fig. 2). Renal impairment ADRs did not influence tepotinib treatment in 22 patients, while they led to treatment interruption (24 patients), dose reduction (20 patients), or discontinuation (seven patients), in the remaining 31 patients (duplicate cases existed). When classified by preferred term, increased blood creatinine was the most common renal ADR (21.8%), followed by impaired renal function (9.5%). Most of the patients with impaired renal function (13/14) reported abnormal laboratory values only, such as a slight increase in blood creatinine.

No instances of QT interval prolongation occurred during the surveillance period.

The incidence of ADRs of safety specifications by age category are presented in Fig. 3a (<65 vs ≥65 years) and Fig. 3b (<75 vs ≥75 years). When evaluated by age categories <65 years and ≥65 years, the incidence of ADRs was 63.0% in patients aged <65 years and 74.2% in patients aged ≥65 years. The incidence of grade ≥3 ADRs was 14.8% and 18.3%, respectively. When assessed using the age categories <75 years and ≥75 years, the incidence of ADRs was 68.3% in patients aged <75 years and 76.9% in patients aged ≥75 years. The incidence of grade ≥3 ADRs was 14.6% and 21.5%, respectively. The incidence of each safety specification is shown in Fig. 3a and b.

There was no notable difference in the incidence of ADRs of safety specifications by ECOG PS (Fig. 3c). The incidence of ADRs was 73.2% in patients with ECOG PS 0–1 and 64.5% in patients with ECOG PS 2–4. The incidence of grade ≥3 ADRs was 16.1% in patients with ECOG PS 0–1 and 22.6% in patients with ECOG PS 2–4. The incidence of each safety specification is shown in Fig. 3c.

**Figure 4:**
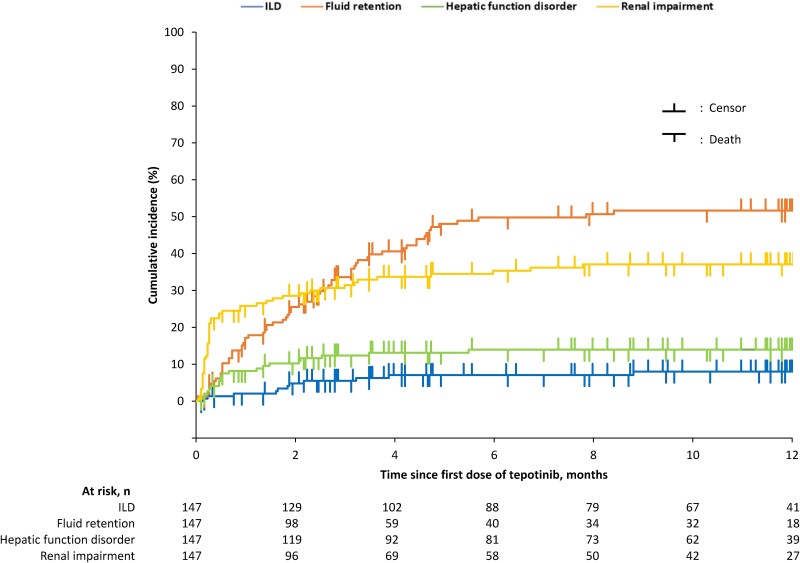
Cumulative incidence rates of adverse drug reactions of safety specifications. ILD, interstitial lung disease; n, number of patients with the event.

Cumulative incidence rates of ILD, fluid retention, hepatic function disorder, and renal impairment ADRs up to 12 months suggested that most ADRs occurred within 4 months, 5 months, 2 months, and 1 month, respectively, after administration of tepotinib (Fig. 4, Supplementary Table S4).

**Table 4:** Tumor response.

	***n* = 147**
Best overall response^a^, n (%)	
CR	4 (2.7)
PR	71 (48.3)
SD	39 (26.5)
PD	18 (12.2)
Could not be adjudicated	1 (0.7)
Unknown	14 (9.5)
ORR, % (95% CI^b^)	51.0 (42.7, 59.3)
DCR, % (95% CI^b^)	77.6 (69.9, 84.0)

CI, confidence interval; CR, complete response; DCR, disease control rate; ORR, objective response rate; PD, progressive disease; PR, partial response; RECIST, Response Evaluation Criteria in Solid Tumors; SD, stable disease.

^a^Best overall response up to week 52, tabulated according to the Japanese version of RECIST, version 1.1.

^b^95% CI was calculated using the Clopper–Pearson method.

### Effectiveness

The best overall response was CR in four patients (2.7%), PR in 71 (48.3%), SD in 39 (26.5%), and PD in 18 (12.2%; Table 4). The ORR was 51.0% (95% CI 42.7, 59.3) and the DCR was 77.6% (95% CI 69.9, 84.0). The median DOR was 8.5 months (95% CI 6.5, 11.3).

**Figure 5:**
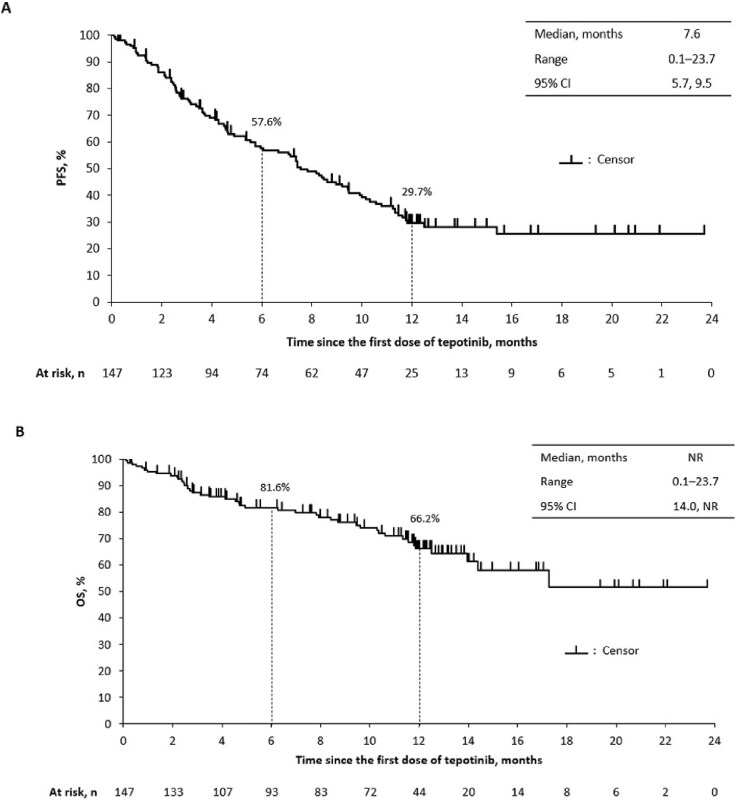
Kaplan–Meier analysis of (a) progression-free survival and (b) overall survival. CI, confidence interval; OS, overall survival; PFS, progression-free survival; n, number of patients; NR, not reached.

The median PFS was 7.6 months (95% CI 5.7, 9.5; Fig. 5a). The PFS rate was 57.6% (95% CI 48.9, 65.3) at 6 months (when 74 patients were still receiving tepotinib) and 29.7% (95% CI 21.9, 37.9) at 12 months (when 25 patients were still receiving tepotinib). The median OS was not reached (Fig. 5b). The OS rate was 81.6% (95% CI 73.9, 87.2) at 6 months and 66.2% (95% CI 56.6, 74.3) at 12 months.

When assessed by patient subgroup (Supplementary Fig. S1), the proportion of patients with an ORR was 59.3% (95% CI 38.8, 77.6) in patients aged <65 years and 49.2% (95% CI 39.9, 58.4) in patients aged ≥65 years. The DCR was 88.9% (95% CI 70.8, 97.6) and 75.0% (95% CI 66.3, 82.5), respectively. In older patients, the proportion with an ORR was 52.4% (95% CI 41.1, 63.6) in those aged <75 years and 49.2% (95% CI 36.6, 61.9) in patients aged ≥75 years. The DCR was 81.7% (95% CI 71.6, 89.4) and 72.3% (95% CI 59.8, 82.7) in the respective age groups. The ORR was 51.8% (95% CI 42.1, 61.3) in patients with ECOG PS 0–1 and 45.2% (95% CI 27.3, 64.0) in patients with ECOG PS 2–4; the corresponding DCR was 82.1% (95% CI 73.8, 88.7) and 61.3% (95% CI 42.2, 78.2), respectively. The ORR was 57.1% (95% CI 47.4, 66.5) in patients with adenocarcinoma, 12.5% (95% CI 0.3, 52.7) in patients with squamous cell carcinoma and 46.2% (95% CI 19.2, 74.9) in patients with sarcomatoid carcinoma. The DCR was 80.4% (95% CI 71.8, 87.3), 62.5% (95% CI 24.5, 91.5) and 61.5% (95% CI 31.6, 86.1), in these respective disease groups.

## Discussion

This PMS evaluated the safety and effectiveness of tepotinib in 147 patients with unresectable, advanced, or recurrent NSCLC with *METex14* skipping alterations in general clinical practice in Japan. The results show that tepotinib was generally well tolerated and efficacious in this patient population. These findings are consistent with those of the VISION study and further validate the clinical benefits of tepotinib in patients with NSCLC and *METex14* skipping alterations [12–14, 19].

The safety profile of tepotinib in the present surveillance was comparable with that shown in the VISION study at the time of approval in Japan [12, 13, 21]. However, the incidences of ILD and renal impairment in this surveillance (7.5% and 36.1%) were higher compared with the overall population of Cohort A in the VISION study (3.8% and 20.0%), and lower compared with Japanese patients enrolled in Cohort A of the VISION study (11.8% and 58.8%). The observed differences between the overall population of Cohort A in the VISION study and this surveillance does not indicate clinically significant differences in trends of occurrence, and are thought to be influenced by differences in patient populations and study design. The incidence of fluid retention (47.6%), hepatic function disorder (13.6%), and QT interval prolongation (0.0%) in this survey were lower or similar compared with that of Cohort A of the VISION study (61.5%, 13.1%, and 1.5%, respectively). Furthermore, the safety profile of tepotinib in this surveillance is consistent with long-term follow-up data from the VISION study [14, 19]. The types of ADRs in this PMS were similar between elderly and non-elderly patients and the incidence of ADRs was not much different for each safety specification when analyzed by age categories <65 vs ≥65 years and <75 vs ≥75 years. The type and incidence of ADRs of safety specifications selected in this PMS was similar in patients with ECOG PS 0–1 and 2–4. Most occurrences of ILD, fluid retention, hepatic function disorder, and renal impairment arose during the first 4, 5, 2, and 1 months of tepotinib treatment, respectively.

Of the four patients who died due to ADRs of safety specifications during this surveillance, three had ILD and one had radiation pneumonitis. Importantly, all four patients presented with active ILD at the start of tepotinib treatment. Three fatal ADRs were initially reported in the VISION study: acute respiratory failure secondary to ILD, severe worsening of dyspnea, and acute hepatic failure [12, 13]. However, the acute hepatic failure event was later revised to a grade 4 ADR [15].

Tepotinib was the first MET inhibitor to receive regulatory approval for NSCLC with *METex14* skipping alterations worldwide (in Japan in 2020) [17, 22]. Other MET inhibitors that have been approved in this indication include capmatinib (in Japan, the United States, and European Union) [23–25] and gumarontinib (in Japan) [26]. Peripheral edema was the most common ADR reported in clinical trials of MET inhibitors in patients with NSCLC and *METex14* skipping alterations and is a known class-effect AE of these drugs [22]. In this surveillance, dose modification and/or diuretic-based treatment tended to resolve peripheral edema compared with no intervention. Most cases of fluid retention occurred within 5 months. The rate of increase of fluid retention within this 5-month period was constant, and the incidence was similar at all time points during this period, which indicates that continuous attention should be given to the incidence of this ADR for 5 months after the initiation of tepotinib. Another ADR often associated with MET inhibitors is increased creatinine levels [22]. MET inhibitors are believed to cause increased creatinine levels by directly influencing creatinine transporters, rather than by inducing renal toxicity [22, 27, 28]. This is supported by the changes in creatinine levels observed in Japanese patients in the VISION study [19], demonstrating the reversibility of creatinine increases upon tepotinib discontinuation. In this surveillance, no accompanying symptoms associated with increased creatinine were reported. ILD is a rare but potentially serious AE that was reported in 2.0%–4.8% of patients in clinical trials of capmatinib and tepotinib [24, 29]. In this study, concurrent complications and pre-existing ILD were considered to have an effect and were risk factors for the development of ILD after the administration of tepotinib. Prior treatment with immune checkpoint inhibitors has been suggested as a factor influencing the occurrence of ILD [30]; however, the results of this surveillance indicated that the incidence of ILD was similar regardless of the presence or absence of prior immune checkpoint inhibitor treatment. Additionally, as a voluntary safety monitoring activity of the sponsor, an expert analysis of the post-marketing ILD ADRs was performed by an independent ILD adjudication committee. The committee pointed out that concurrent complications and pre-existing ILD or interstitial changes in the lungs at baseline may be a factor in poor prognosis, however no specific clinical or radiological features were observed with tepotinib-induced ILD in Japanese patients [31]. It has been recommended that patients receiving MET inhibitors for the treatment of NSCLC with *METex14* skipping alterations should be carefully monitored for peripheral edema, increased creatinine, and ILD events, and undergo regular liver and renal function testing [22].

The effectiveness of tepotinib in the present surveillance was consistent with that shown in the VISION study, with similar ORR (51.0%) and DCR (77.6%) in this PMS vs 51.4% and 76.0%, respectively [12–14]; the ORR and DCR in the Japanese subset of patients in the VISION study was 60.5% and 78.9%, respectively [19]. However, because this PMS did not specify the timing of tumor assessments, PFS and DOR could not be compared with those reported in the VISION study [12, 13]. Subgroups that were excluded from the VISION study criteria (ECOG PS 2–4, beyond fourth-line treatment) accounted for 21.1% and 22.4% of patients, respectively, in the current surveillance; nevertheless, based on the results of this PMS, tepotinib demonstrated a certain level of effectiveness in this real-world setting. As shown in Supplementary Fig. S1, it is notable that clinically relevant effectiveness was observed in patients with ECOG PS 2–4 and those who received ≥4 lines of treatment, with ORR of 45.2% and 51.5%, respectively, and DCR of 61.3% and 75.8%, respectively. The present surveillance was designed to specifically include patients with unresectable, advanced, or recurrent NSCLC with *METex14* skipping alterations who were treated with tepotinib in Japan. Therefore, it provides an accurate reflection of the safety and effectiveness of tepotinib in this patient population in the general clinical practice setting.

This PMS was designed to collect data on five specific ADRs only; therefore information on ADRs other than these five specific safety specifications were not collected. However, ADRs reported outside of this PMS were evaluated as part of post-marketing pharmacovigilance activities, and no new safety signals have been identified.

Some limitations of this PMS include its non-interventional observational design with no control group and that data were not assessed by an independent review committee. Source data verification was not required as per GPSP ordinance and was not performed against medical records. Results may be biased due to investigator assessments and it may be difficult to clarify whether the obtained results were caused by tepotinib treatment. The incidence of ADRs may have been affected by the duration of tepotinib administration. In this context, elderly patients or those with poor PS typically had shorter treatment durations, which could be a confounding factor. In addition, the observation period was limited to a maximum of 52 weeks from the start of tepotinib administration, and ADRs of safety specifications that occurred after 52 weeks were not collected. Furthermore, the estimates of time-dependent effectiveness (DOR, PFS, and OS) may have limited reliability.

In conclusion, tepotinib was generally well tolerated and effective in Japanese patients with unresectable, advanced, or recurrent NSCLC harboring *METex14* skipping alterations. These data on the use of tepotinib in general clinical practice support the findings of the VISION study, showing a comparable risk–benefit profile.

## Supplementary Material

Kato_Supplemental_07May25_not_tracked_hyaf099

## Data Availability

Any requests for data by qualified scientific and medical researchers for legitimate research purposes will be subject to Merck’s (CrossRef Funder ID: 10.13039/100009945) Data Sharing Policy. All requests should be submitted in writing to Merck’s data sharing portal (https://www.merckgroup.com/en/research/our-approach-to-research-and-development/healthcare/clinical-trials/commitment-responsible-data-sharing.html). When Merck has a co-research, co-development, co-marketing, or co-promotion agreement, or when the product has been out-licensed, the responsibility for disclosure might be dependent on the agreement between parties. Under these circumstances, Merck will endeavor to gain agreement to share data in response to requests.

## Notes

Conflict of interest: T.K. has been an advisor or consultant for AbbVie, AstraZeneca, Boehringer Ingelheim, Daiichi Sankyo, Johnson & Johnson, Merck Biopharma Co, Ltd, Tokyo, Japan, an affiliate of Merck KGaA, MSD, and Novocure; a speaker or lecturer for Amgen, Bristol Myers Squibb, Chugai, Eli Lilly, Novartis, Ono, Pfizer, and Takeda; and has received grants for clinical research from AbbVie, Amgen, ArriVent, AstraZeneca, Bayer, BeiGene, Blueprint Medicines, Boehringer Ingelheim, Bristol Myers Squibb, Chugai, Daiichi Sankyo, Eli Lilly, Gilead, GlaxoSmithKline, Haihe, Johnson & Johnson, Merck Biopharma Co, Ltd, Tokyo, Japan, an affiliate of Merck KGaA, MSD, Novartis, Novocure, Pfizer, Regeneron, and Takeda.

T.O., M.S., R.K., B.S., and E.H., are employees of Merck Biopharma Co, Ltd, Tokyo, Japan, an affiliate of Merck KGaA.

K.N. reports grants or contracts from Amgen Inc, MSD K.K., Ono Pharmaceutical Co, Ltd, Nippon Boehringer Ingelheim Co, Ltd, Novartis Pharma K.K., Pfizer Japan Inc, Bristol Myers Squibb Company, Eli Lilly Japan K.K., Chugai Pharmaceutical Co, Ltd, Daiichi Sankyo Co, Ltd, AstraZeneca K.K., Parexel International Corp, PRA Health Sciences, EPS Corporation, Kissei Pharmaceutical Co, Ltd, Taiho Pharmaceutical Co, Ltd, PPD-SNBL K.K., SymBio Pharmaceuticals Ltd, IQVIA Services Japan K.K., Syneos Health Clinical K.K., Nippon Kayaku Co, Ltd, EP-CRSU Co, Ltd, Mebix, Inc, Janssen Pharmaceutical K.K., Astellas Pharma Inc, Bayer Yakuhin, Ltd, Eisai Co, Ltd, Mochida Pharmaceutical Co, Ltd, LabCorp Development Japan K.K. (Covance Japan Inc), Japan Clinical Research Operations, Takeda Pharmaceutical Co, Ltd, GlaxoSmithKline K.K., Sanofi K.K., Sysmex Corporation, Medical Research Support, Otsuka Pharmaceutical Co, Ltd, SRL Inc, Pfizer R&D Japan G.K., CMIC Co, Ltd, Eisai Inc, Shionogi & Co, Ltd, Kobayashi Pharmaceutical Co, Ltd, and Ascent Development Services; consulting fees from Eli Lilly Japan K.K. and Ono Pharmaceutical Co, Ltd; payment or honoraria from Ono Pharmaceutical Co, Ltd, Amgen Inc, Nippon Kayaku Co, Ltd, AstraZeneca K.K., Chugai Pharmaceutical Co, Ltd, Eli Lilly Japan K.K., MSD K.K., Pfizer Japan Inc, Nippon Boehringer Ingelheim Co, Ltd, Taiho Pharmaceutical Co, Ltd, Bayer Yakuhin, Ltd, CMIC ShiftZero K.K., Life Technologies Japan Ltd, Neo Communication, Daiichi Sankyo Co, Ltd, Incyte Biosciences Japan, Merck Biopharma Co, Ltd, Tokyo, Japan, an affiliate of Merck KGaA, Kyowa Kirin Co, Ltd, Takeda Pharmaceutical Co, Ltd, 3H Clinical Trial Inc, Care Net, Inc, Medical Review Co, Ltd, Medical Mobile Communications Co, Ltd, Yodosha Co, Ltd, Nikkei Business Publications, Inc, Japan Clinical Research Operations, CMIC Co, Ltd, Novartis Pharma K.K., Taiyo Pharma Co, Ltd, Janssen Pharmaceutical K.K., and Bristol Myers Squibb K.K.; and patents with Daiichi Sankyo Co, Ltd.
