# Supplementary material for: Safety and effectiveness of tepotinib in patients with unresectable, advanced or recurrent non-small cell lung cancer with METex14 skipping alterations: post-marketing surveillance in Japan
Source: Jpn J Clin Oncol. 2025 Jun 20;55(10):1152–61. doi: 10.1093/jjco/hyaf099 (PMC12501971; doi:10.1093/jjco/hyaf099)
Supplement: Kato_Supplemental_07May25_not_tracked_hyaf099 [file kato_supplemental_07may25_not_tracked_hyaf099.docx]

**Safety and effectiveness of tepotinib in patients with unresectable, advanced or recurrent non-small cell lung cancer with *METex14* skipping alterations: post-marketing surveillance in Japan**

**Supplementary materials**

**Authors:** Terufumi Kato^1^, Tatsuya Ogura^2^, Masashi Sato^3^, Risa Kojima^4^, Bingbing Song^5^, Eisuke Horii^6^, Kazuhiko Nakagawa^7^

**Affiliations:** ^1^Department of Thoracic Oncology, Kanagawa Cancer Center, Yokohama, Japan; ^2^PMS Planning & Strategy, Merck Biopharma Co., Ltd., Tokyo, Japan, an affiliate of Merck KGaA; ^3^Global Research & Development, Clinical Measurement Sciences, Biostatistics, Merck Biopharma Co., Ltd., Tokyo, Japan, an affiliate of Merck KGaA; ^4^Global Patient Safety Japan, Merck Biopharma Co., Ltd., Tokyo, Japan, an affiliate of Merck KGaA; ^5^Global Development Operations Japan, R&D, Merck Biopharma Co., Ltd., Tokyo, Japan, an affiliate of Merck KGaA; ^6^Oncology Medical Affairs, Merck Biopharma Co., Ltd., Tokyo, Japan, an affiliate of Merck KGaA; ^7^Department of Medical Oncology, Kindai University Faculty of Medicine, Osaka, Japan

**Corresponding author:** Terufumi Kato, Department of Thoracic Oncology, Kanagawa Cancer Center, Yokohama, Japan. Tel: 045-520-2222; email: katote@kcch.jp

## **Supplementary Table S1.**

Patient characteristics according to age.

| **Characteristic** | **<65 years old (n=27)** | **≥65 years  (n=120)** | **<75 years  (n=82)** | **≥75 years  (n=65)** |
| --- | --- | --- | --- | --- |
| Age, years, median (range) | 62.0 (42–64) | 76.0 (65–90) | 68.0 (42–74) | 80.0 (75–90) |
| Age, n (%) |  |  |  |  |
| <65 years | 27 (100.0) | – | 27 (32.9) | – |
| 65 to <75 years | – | 55 (45.8) | 55 (67.1) | – |
| 75 to <85 years | – | 50 (41.7) | – | 50 (76.9) |
| ≥85 years | – | 15 (12.5) | – | 15 (23.1) |
| Sex, n (%) |  |  |  |  |
| Male | 12 (44.4) | 67 (55.8) | 41 (50.0) | 38 (58.5) |
| Female | 15 (55.6) | 53 (44.2) | 41 (50.0) | 27 (41.5) |
| Smoking history, n (%) |  |  |  |  |
| Never | 15 (55.6) | 54 (45.0) | 39 (47.6) | 30 (46.2) |
| Current | – | 7 (5.8) | 3 (3.7) | 4 (6.2) |
| Former | 12 (44.4) | 59 (49.2) | 40 (48.8) | 31 (47.7) |
| ECOG PS, n (%) |  |  |  |  |
| 0 | 11 (40.7) | 25 (20.8) | 22 (26.8) | 14 (21.5) |
| 1 | 11 (40.7) | 65 (54.2) | 43 (52.4) | 33 (50.8) |
| 2 | – | 17 (14.2) | 8 (9.8) | 9 (13.8) |
| 3 | 4 (14.8) | 9 (7.5) | 7 (8.5) | 6 (9.2) |
| 4 | – | 1 (0.8) | – | 1 (1.5) |
| Unknown | 1 (3.7) | 3 (2.5) | 2 (2.4) | 2 (3.1) |
| Histology, n (%) |  |  |  |  |
| Adenocarcinoma | 22 (81.5) | 90 (75.0) | 62 (75.6) | 50 (76.9) |
| Sarcomatoid carcinoma | 1 (3.7) | 12 (10.0) | 4 (4.9) | 9 (13.8) |
| Squamous cell carcinoma | 1 (3.7) | 7 (5.8) | 6 (7.3) | 2 (3.1) |
| Other^a^ | 3 (11.1) | 11 (9.2) | 10 (12.2) | 4 (6.2) |
| Treatment line, n (%) |  |  |  |  |
| First | 11 (40.7) | 60 (50.0) | 33 (40.2) | 38 (58.5) |
| Second | 4 (14.8) | 26 (21.7) | 16 (19.5) | 14 (21.5) |
| Third | 4 (14.8) | 9 (7.5) | 9 (11.0) | 4 (6.2) |
| Fourth or later | 8 (29.6) | 25 (20.8) | 24 (29.3) | 9 (13.8) |
| Prior treatment history of NSCLC^b^, n (%) |  |  |  |  |
| Chemotherapy | 18 (66.7) | 57 (47.5) | 51 (62.2) | 24 (36.9) |
| ICI | 13 (48.1) | 44 (36.7) | 41 (50.0) | 16 (24.6) |
| Crizotinib | 1 (3.7) | 4 (3.3) | 4 (4.9) | 1 (1.5) |
| Other^c^ | 8 (29.6) | 15 (12.5) | 19 (23.2) | 4 (6.2) |
| Concurrent complications^b^, n (%) |  |  |  |  |
| Renal impairment | 6 (22.2) | 22 (18.3) | 15 (18.3) | 13 (20.0) |
| Hepatic function disorder | 3 (11.1) | 6 (5.0) | 7 (8.5) | 2 (3.1) |
| Allergy | 2 (7.4) | 6 (5.0) | 6 (7.3) | 2 (3.1) |
| ILD | 1 (3.7) | 6 (5.0) | 5 (6.1) | 2 (3.1) |
| PD-L1 status^d^, n (%) |  |  |  |  |
| Not tested | 3 (11.1) | 11 (9.2) | 6 (7.3) | 8 (12.3) |
| Tested | 24 (88.9) | 109 (90.8) | 76 (92.7) | 57 (87.7) |
| Positive | 17 (70.8) | 89 (81.7) | 56 (73.7) | 50 (87.7) |
| Negative | 7 (29.2) | 20 (18.3) | 20 (26.3) | 7 (12.3) |

^a^Includes non-small cell carcinoma, not otherwise specified and adenosquamous carcinoma. ^b^Patients could have multiple conditions. ^c^Includes other oncology drugs, gefitinib, osimertinib mesylate, bevacizumab and ramucirumab. ^d^Calculated on a reported basis, regardless of the positivity rate.
ECOG PS, Eastern Cooperative Oncology Group performance status; ICI, immune checkpoint inhibitor; ILD, interstitial lung disease; NSCLC, non-small cell lung cancer; PD-L1, programmed death-ligand 1.

## **Supplementary Table S2.**

Patient characteristics according to Eastern Cooperative Oncology Group performance status.

| **Characteristic** | **ECOG PS 0–1 (n=112)** | **ECOG PS 2–4  (n=31)** |
| --- | --- | --- |
| Age, years, median (range) | 72.0 (42–89) | 76.0 (50–90) |
| Age, n (%) |  |  |
| <65 years | 22 (19.6) | 4 (12.9) |
| 65 to <75 years | 43 (38.4) | 11 (35.5) |
| 75 to <85 years | 35 (31.3) | 13 (41.9) |
| ≥85 years | 12 (10.7) | 3 (9.7) |
| Sex, n (%) |  |  |
| Male | 57 (50.9) | 20 (64.5) |
| Female | 55 (49.1) | 11 (35.5) |
| Smoking history, n (%) |  |  |
| Never | 55 (49.1) | 12 (38.7) |
| Current | 5 (4.5) | 1 (3.2) |
| Former | 52 (46.4) | 18 (58.1) |
| ECOG PS, n (%) |  |  |
| 0 | 36 (32.1) | – |
| 1 | 76 (67.9) | – |
| 2 | – | 17 (54.8) |
| 3 | – | 13 (41.9) |
| 4 | – | 1 (3.2) |
| Histology, n (%) |  |  |
| Adenocarcinoma | 84 (75.0) | 24 (77.4) |
| Sarcomatoid carcinoma | 11 (9.8) | 2 (6.5) |
| Squamous cell carcinoma | 5 (4.5) | 3 (9.7) |
| Other^a^ | 12 (10.7) | 2 (6.5) |
| Treatment line, n (%) |  |  |
| First | 55 (49.1) | 15 (48.4) |
| Second | 22 (19.6) | 7 (22.6) |
| Third | 9 (8.0) | 3 (9.7) |
| Fourth or later | 26 (23.2) | 6 (19.4) |
| Prior treatment history of NSCLC^b^, n (%) |  |  |
| Chemotherapy | 57 (50.9) | 15 (48.4) |
| ICI | 43 (38.4) | 11 (35.5) |
| Crizotinib | 5 (4.5) | – |
| Other^c^ | 20 (17.9) | 3 (9.7) |
| Concurrent complications^b^, n (%) |  |  |
| Renal impairment | 20 (17.9) | 8 (25.8) |
| Hepatic function disorder | 6 (5.4) | 3 (9.7) |
| Allergy | 5 (4.5) | 2 (6.5) |
| ILD | 4 (3.6) | 2 (6.5) |
| PD-L1 status^d^, n (%) |  |  |
| Not tested | 10 (8.9) | 4 (12.9) |
| Tested | 102 (91.1) | 27 (87.1) |
| Positive | 84 (82.4) | 20 (74.1) |
| Negative | 18 (17.6) | 7 (25.9) |

^a^Includes non-small cell carcinoma, not otherwise specified and adenosquamous carcinoma. ^b^Patients could have multiple conditions. ^c^Includes other oncology drugs, gefitinib, osimertinib mesylate, bevacizumab and ramucirumab. ^d^Calculated on a reported basis, regardless of the positivity rate.
ECOG PS, Eastern Cooperative Oncology Group performance status; ICI, immune checkpoint inhibitor; ILD, interstitial lung disease; NSCLC, non-small cell lung cancer; PD-L1, programmed death-ligand 1.

## **Supplementary Table S3.**

1. Incidence of serious and non-serious adverse drug reactions of safety specifications.

| **ADR, n (%)** | **N=147** | |
| --- | --- | --- |
|  | **Serious** | **Non-serious** |
| ILD | 7 (4.8) | 4 (2.7) |
| ILD | 5 (3.4) | 3 (2.0) |
| Pneumonitis | 1 (0.7) | 1 (0.7) |
| Radiation pneumonitis | 1 (0.7) | – |
| Fluid retention | 9 (6.1) | 61 (41.5) |
| Hypoalbuminemia | – | 1 (0.7) |
| Periorbital edema | – | 1 (0.7) |
| Pleural effusion | 2 (1.4) | 5 (3.4) |
| Systemic edema | 3 (2.0) | 3 (2.0) |
| Peripheral edema | 4 (2.7) | 53 (36.1) |
| Localized edema | – | 1 (0.7) |
| Hepatic function disorder | 2 (1.4) | 18 (12.2) |
| Ascites | 1 (0.7) | – |
| Abnormal hepatic function | – | 9 (6.1) |
| Hepatobiliary disease | 1 (0.7) | – |
| Drug-induced liver injury | – | 1 (0.7) |
| ALT increased | – | 7 (4.8) |
| AST increased | – | 6 (4.1) |
| Renal impairment | 3 (2.0) | 50 (34.0) |
| Renal disorder | – | 5 (3.4) |
| Impaired renal function | 1 (0.7) | 13 (8.8) |
| Acute kidney injury | 1 (0.7) | 2 (1.4) |
| Blood creatinine increased | 2 (1.4) | 30 (20.4) |

ADRs of safety specifications were classified according to MedDRA/J, version 25.1.
ADR, adverse drug reaction; ALT, alanine aminotransferase; AST, aspartate aminotransferase; ILD, interstitial lung disease; MedDRA/J, Japanese version of Medical Dictionary for Regulatory Activities.

## **Supplementary Table S4.**

Cumulative incidence of adverse drug reactions of safety specifications.

| **Timepoint** | **ILD** | | | **Fluid retention** | | | **Hepatic function disorder** | | | **Renal impairment** | | |
| --- | --- | --- | --- | --- | --- | --- | --- | --- | --- | --- | --- | --- |
|  | **At risk, n** | **ADR, n** | **Cumulative incidence,  % (95% CI)** | **At risk, n** | **ADR, n** | **Cumulative incidence, % (95% CI)** | **At risk, n** | **ADR, n** | **Cumulative incidence, % (95% CI)** | **At risk, n** | **ADRs, n** | **Cumulative incidence, % (95% CI)** |
| 1 month | 137 | 3 | 2.1 (0.6, 5.4) | 113 | 25 | 17.2 (11.6, 23.8) | 125 | 13 | 8.9 (5.0, 14.2) | 102 | 38 | 25.9 (19.1, 33.1) |
| 2 months | 129 | 4 | 4.8 (2.1, 9.2) | 98 | 12 | 25.5 (18.7, 32.9) | 118 | 3 | 10.9 (6.5, 16.6) | 96 | 4 | 28.6 (21.5, 36.0) |
| 3 months | 113 | 1 | 5.5 (2.6, 10.1) | 72 | 12 | 34.3 (26.6, 42.2) | 99 | 3 | 13.1 (8.2, 19.1) | 78 | 4 | 31.4 (24.1, 39.1) |
| 4 months | 102 | 2 | 7.1 (3.6, 12.1) | 58 | 9 | 41.3 (33.0, 49.4) | 91 | 1 | 13.8 (8.8, 20.0) | 69 | 3 | 33.7 (26.1, 41.4) |
| 5 months | 90 | 0 | 7.1 (3.6, 12.1) | 42 | 9 | 48.8 (40.1, 57.0) | 82 | 0 | 13.8 (8.8, 20.0) | 60 | 1 | 34.5 (26.8, 42.3) |
| 6 months | 88 | 0 | 7.1 (3.6, 12.1) | 39 | 2 | 50.5 (41.7, 58.7) | 80 | 1 | 14.7 (9.4, 21.0) | 58 | 1 | 35.3 (27.6, 43.2) |
| 7 months | 84 | 0 | 7.1 (3.6, 12.1) | 38 | 0 | 50.5 (41.7, 58.7) | 78 | 0 | 14.7 (9.4, 21.0) | 55 | 1 | 36.2 (28.3, 44.1) |
| 8 months | 79 | 0 | 7.1 (3.6, 12.1) | 33 | 1 | 51.5 (42.5, 59.6) | 72 | 0 | 14.7 (9.4, 21.0) | 50 | 1 | 37.1 (29.1, 45.0) |
| 9 months | 73 | 1 | 8.0 (4.2, 13.4) | 31 | 1 | 52.4 (43.5, 60.6) | 68 | 0 | 14.7 (9.4, 21.0) | 47 | 0 | 37.1 (29.1, 45.0) |
| 10 months | 67 | 0 | 8.0 (4.2, 13.4) | 31 | 0 | 52.4 (43.5, 60.6) | 62 | 0 | 14.7 (9.4, 21.0) | 42 | 0 | 37.1 (29.1, 45.0) |
| 11 months | 64 | 0 | 8.0 (4.2, 13.4) | 29 | 0 | 52.4 (43.5, 60.6) | 57 | 0 | 14.7 (9.4, 21.0) | 38 | 0 | 37.1 (29.1, 45.0) |
| 12 months | 41 | 0 | 8.0 (4.2, 13.4) | 18 | 0 | 52.4 (43.5, 60.6) | 39 | 0 | 14.7 (9.4, 21.0) | 27 | 0 | 37.1 (29.1, 45.0) |

ADR, adverse drug reaction; CI, confidence interval; ILD, interstitial lung disease.

## **Supplementary Figure S1.**

Subgroup analysis of objective response rate and disease control rate.


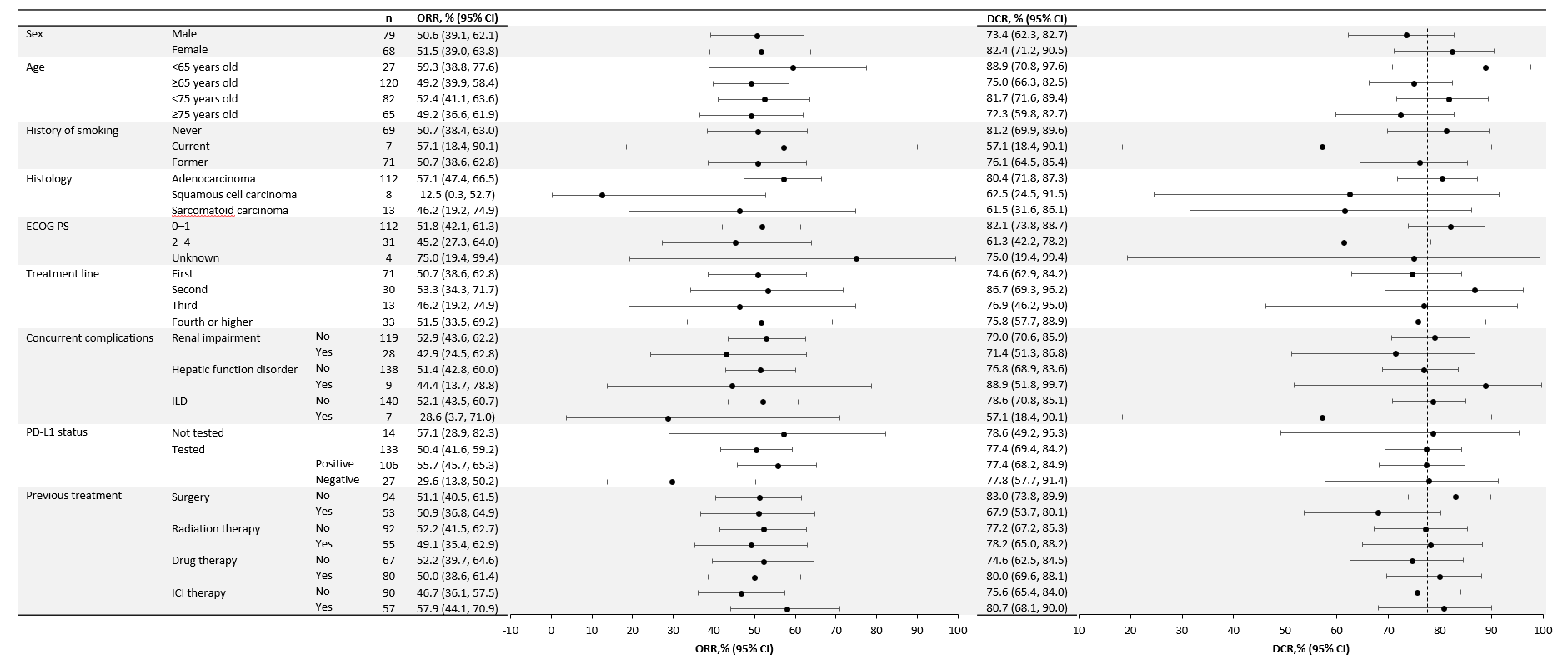
CI, confidence interval; DCR, disease control rate; ECOG PS, Eastern Cooperative Oncology Group performance status; ICI, immune checkpoint inhibitor; ILD, interstitial lung disease; ORR, objective response rate; PD-L1, programmed death-ligand 1.
